# Coumarins of Lovage Roots (*Levisticum officinale* W.D.J.Koch): LC-MS Profile, Quantification, and Stability during Postharvest Storage

**DOI:** 10.3390/metabo13010003

**Published:** 2022-12-20

**Authors:** Daniil N. Olennikov

**Affiliations:** Laboratory of Biomedical Research, Institute of General and Experimental Biology, Siberian Division of Russian Academy of Science, Sakh’yanovoy Street 6, 670047 Ulan-Ude, Russia; olennikovdn@mail.ru; Tel.: +7-90-2160-0627

**Keywords:** lovage, *Levisticum officinale*, coumarins, liquid chromatography–mass spectrometry, postharvest storage

## Abstract

Lovage (*Levisticum officinale* W.D.J. Koch) is a known aromatic apiaceous species that is widely used as a culinary and medicinal plant. Traditionally, more scientific attention has been paid to lovage volatiles, while other groups of compounds have been underutilized. In this study, metabolites of fresh lovage roots were investigated by liquid chromatography–mass spectrometry, and 25 compounds were identified, including coumarins as basic components and minor hydroxycinnamates; most were detected for the first time in the plant. Four major coumarins (including apterin, xanthotoxin, isopimpinellin, and pimpinellin) were successfully separated by a validated HPLC–PDA method, and the fresh roots of seven lovage cultivars as well as the dry roots of commercial lovage were quantified. The coumarin content deviation was 1.7–2.9 mg/g in the fresh roots and 15–24 mg/g in the dry roots. A variation in the coumarin level was found during storage of the fresh lovage roots at chill and room temperatures, while storage of the dried roots at room temperature showed the lowest loss of target compounds. This new information about the metabolites of lovage indicates the prospects of the plant roots as a source of dietary coumarins.

## 1. Introduction

Lovage (*Levisticum officinale* W.D.J. Koch; syn. *Angelica levisticum* (L.) All., *Ligusticum levisticum* L., and *Selinum levisticum* (L.) E.H.L. Krause)) is a single species of the *Levisticum* Hill genus included in the Apiaceae family. Lovage grows natively in Southwest Asia (Hazaran Mountain, Kerman province, Iran) and southern Europe and has been cultivated globally for a long time as a food plant and a source of spicy greens [1]. The parts of the plant that are used include the underground organs, the aboveground part, as well as seeds, which are typical for some food species of Apiaceae (celery, parsley, parsnip), in which greens, roots, and fruits have culinary value [2]. Lovage leaves are used as spicy salad greens because they have a characteristic odor close to the odor of celery and parsley, and the roots are used as a cooked or fresh vegetable [3]. Despite the lower popularity of lovage compared to related species, interest in the study of the component composition arose in the late 19th and early 20th centuries, as evidenced by the data on the isolation of α-terpineol, cineol, and myristic acid from the roots [4]. Soon after, Naves (1943) discovered and characterized four phtalides (butyl phtalide, butyl dihydrophtalide, butyl tetrahydrophtalide, and butylidene phthalide) [4]. Subsequently, approximately 20 representatives of the phtalides group were isolated from the plant [5,6,7,8,9,10,11,12,13,14,15,16] (Table 1). Further investigation led to detection of polyynes, mono- and sesquiterpenes, phenolics, and other groups [17,18,19,20,21,22,23,24,25,26,27,28,29,30]. 

Because all organs of the plant have a pronounced odor, the main studies of *L. officinale* have been focused on the study of the spicy-flavored components, which allowed to determine in detail the composition of the essential oil and volatile fractions from all organs of the plant, including roots [5,6,8,9,11,18,19,20,21,22,23,24], fruits [5,6,11,23,25], leaves [5,6,11,12,18,21,26], stems [5,6,11,12,18], and flowers [23,24,25,27]. Very little attention has been paid to the study of other groups of extractives. In particular, the presence of some simple phenols (eugenol, carvacrol, methyl salicylate, cuminaldehyde, estragole, and pentyl benzene) is known in volatile extracts of roots [4,11,21], fruits [6,25], and leaves [21], and the presence of hydroxycinnamates (derivatives of caffeic acid, ferulic acid, etc.) [10,12,14,15,29,30,31], coumarins [4,14,15,28], and flavonoids [12,14,15,29] is known in alcohol-derived extracts of the roots and leaves. Trustworthy information regarding fresh lovage roots is extremely scarce, partially owing to the difficulties of practical work with fresh tissue. This detrimental attitude towards the study of spice plants is unfortunately typical, leading to a profound lack of knowledge about the metabolome of this food species.

One of the poorly studied groups of phytocomponents characteristic of the food species of Apiaceae is the coumarin group, a group of phenolic α-pyrone compounds, which are well-studied for non-food members of the family but are still underutilized in regular products [32]. Coumarins of Apiaceae include simple coumarins (umbelliferon, scopoletin, esculetin) [33]; furanocoumarins (psoralen, bergapten, etc.) have been detected in celery [34], dill [35], and carrot [32]; pyranocoumarins (visnadin, dihydrosamidin, etc.) are typical for medical species such as *Angelica* [36], *Peucedanum* [37], and *Phlojodicarpus* [38,39]. Known data on coumarins of *L. officinale* are limited, and include information about pyranocoumarins psoralen, 5-methoxypsoralen, and imperatorin from fruits [28], apterin from the leaves [14,15], and bergapten from roots [4]. This information cannot be considered complete and needs additional study of the componential profile and quantification data of *L. officinale* coumarins. Additionally, furanocoumarins are bioactive metabolites with proven antivirus [40], antiallergic [41], antidiabetic [42], antidepressive [43], anticancer [44], and anti-inflammatory potential [45]. Thus, furanocoumarin-containing foods may be promising functional products.

Apiaceous vegetables (e.g., carrot, celery, and parsley) have a culinary application as fresh roots; therefore, owing to the limited postharvest shelf life, the study of chemical changes deserves special attention to reduce losses. In particular, it is known that the process of storing reduces the content of carotenes, phenolics, and ascorbic acid in fresh carrot roots [46,47], and chill storage allows to slow down destructive processes [48]. Twenty-days of storage of fresh celery roots resulted in the decrease or increase in the content of chlorogenic acids, depending on the variety [49], while the ascorbic acid content reduced after six days of postharvest refrigerated storage [50]. There is no information about postharvest stability of coumarins in lovage and other apiaceous species despite their obligate presence in root products.

As part of the ongoing study of Apiaceae coumarins [36,37,38,39], high-performance liquid chromatography with photodiode array detection with electrospray ionization triple quadrupole mass spectrometric detection (HPLC–PDA–ESI–TQ–MS) was applied for phenolic metabolite profiling of the fresh roots of lovage (*L. officinale*), followed by the quantification of the principal components by rapid HPLC–PDA of the fresh roots of seven lovage cultivars and dry commercial products, and the postharvest changes of coumarins in the lovage roots were studied.

## 2. Materials and Methods

### 2.1. Plant Material and Chemicals

Cultivated samples of *Levisticum officinale* roots were harvested in Buryat Fruit and Plant Nursery located in the vicinity of Ulan-Ude (Russia) using authenticated seeds of lovage (cv. Amur, Don Juan, Heracles, Lider, Magnus, Preobrazhenskii, Udalets) purchased in the National Seed Repository (Moscow, Russia). All plants were authenticated by Prof. N.I. Kashchenko (IGEB SB RAS, Ulan-Ude, Russia). The fresh roots were conditioned in plastic boxes and transported to the laboratory at 4 °C within 2–3 h. The reference standards were purchased from Anexib Chemicals (Richmond Hill, Ontario, Canada): peucedanin (≥95%; No P173001); Biopurity Phytochemicals Ltd. (Chengdu, Sichuan, China): skimmin (≥98%; No BP1316); ChemFaces (Wuhan, Hubei, China): apterin (≥98%; No CFN95005), 5-*O*-caffeoylquinic acid (≥98%; No. 94419), 1,3-di-*O*-caffeoylquinic acid (≥98%; No. D8196), 3,4-di-*O*-caffeoylquinic acid (≥90%; No. SMB00224), 3,5-di-*O*-caffeoylquinic acid, 4,5-di-*O*-caffeoylquinic acid (≥85%; No. SMB00221), cichoriin (≥98%; No CFN95196), isobergapten (≥98%; No CFN90231), 5-*O*-feruloylquinic acid (≥98%; No CFN92889); MedChemExpress, Monmouth Junction, NJ, USA: angelicin (≥98%; No HY-N0763), apiosylskimmin (≥98%; No HY-N2356), isoimperatorin (≥98%; No HY-N0286), pimpinellin (≥98%; No HY-N0438); Sigma-Aldrich (St. Louis, MO, USA): bergapten (≥99%; No 69664), esculin (≥95%; No Y0001612), esculetin (≥98%; No 246573), isopimpinellin (≥95%; No 61419), umbelliferone (≥99%; No H24003), xanthotoxin (≥98%; No 56448); Selleck Chemicals (Houston, TX, USA): imperatorin (≥98%; No S380901), osthole (≥98%; No S2337), and psoralen (≥98%; No S4737).

### 2.2. Plant Extracts Preparation

Fresh roots of 3 y.o. lovage plants (25–30 cm long) were homogenized by X-1740 homogenizer (Goldleaf Scientific, Riverside, CA, USA), and a portion of homogenate (5 g) was treated by 45 mL of methanol and sonicated twice (ultrasonic bath, 20 min, 50 °C, ultrasound power 100 W, frequency 35 kHz). Dry lovage roots were ground in laboratory grinder KM-100 (MRC group, Harlow, Essex, UK) till particle size 0.125 μm, and 1-g sample was extracted by 50 mL of methanol with double sonication (ultrasonic bath, 40 min, 50 °C, ultrasound power 100 W, frequency 35 kHz). Methanolic extract (after fresh or dry tissue extraction) was filtered through 0.22-μm syringe filters into a measuring flask (100 mL) and the final volume was filled up to 100 mL by methanol. The resultant extract was stored at 2 °C before analysis.

### 2.3. High-Performance Liquid Chromatography with Photodiode Array Detection and Electrospray Ionization Triple Quadrupole Mass Spectrometric Detection (HPLC-PDA-ESI-TQ-MS) Metabolite Profiling

Lovage roots metabolite profiling was performed by HPLC-PDA-ESI-TQ-MS assay on the liquid chromatograph LC-20 Prominence coupled with photodiode array detector SPD-M30A (wavelength range 200–600 nm), triple-quadrupole mass spectrometer LCMS 8050 (all Shimadzu, Columbia, MD, USA) and ProntoSIL 120-5 C18 AQ column (1 mm × 50 mm, 1 μm; Knauer, Berlin, Germany). The gradient elution used eluents A (1% acetic acid in water) and B (1% acetic acid in acetonitrile) and the gradient program (%B): 0–4 min 5–100%, 4–5 min 100%, 5–6 min 100–5%, and 6–7 min 5%. The injection volume was 0.5 μL and the flow rate was 500 μL/min. Ultraviolet spectra were recorded in a spectral range 200–600 nm. Electrospray ionization triple quadrupole mass spectrometric detection used temperature 300 °C in the ESI interface, 250 °C in the desolvation line, and 400 °C in the heat block. The nebulizing gas (N_2_) flow value was 3 L/min, heating gas (air)—10 L/min, and collision-induced dissociation gas (Ar)—0.3 mL/min. The source voltage of mass spectra was 3 kV, collision energy was +10–+25 eV (positive ionization), and the scanning range was *m*/*z* 80–1900. LabSolution’s workstation software managed the LC-MS system. The integrated analysis of retention time, ultraviolet and mass spectra data after comparison with the inner LC-MS library, reference standards and the literature data were used for the identification of metabolites.

### 2.4. HPLC-PDA-MS Metabolite Quantification

To quantify four coumarins (apterin, xanthotoxin, isopimpinellin, pimpinellin) in plant extracts, the HPLC-PDA-ESI-TQ-MS separation and detection conditions were used (Section 2.3). Reference standards were separately weighed (10 mg), dissolved in the methanol in volumetric flasks (10 mL), and the stock solution (1000 µg/mL) was used to prepare the calibration solutions (1–100 µg/mL). After the separation, PDA data were used to create ‘concentration–PDA peak area’ correlation. Correlation coefficient (r^2^), standard deviation (S_YX_), limit of detection (LOD), limit of quantification (LOQ), and linear range were calculated in Advanced Grapher 2.2 (Alentum Software Inc., Ramat-Gan, Israel) using calibration curve data. Values of intra-day and inter-day precisions and recovery of spiked sample were determined as described early [38]. Three HPLC runs were sufficient for the quantitative analyses, and the results were expressed as mean values ± standard deviation (S.D.).

### 2.5. Lovage Roots Storage Experiment

Six and five portions of the fresh lovage samples (10 roots, approx. equal; cv. Lider) were deposited into the individual polystyrene bags (2 L) and incubated at (1) 1 °C (6 months) or (2) at 20 °C (14 days), respectively, in a ventilated MK 53 thermostat (BINDER GmbH, Tuttlingen, Germany). Five roots of stored samples were taken out for analysis (1) every month or (2) at 1, 3, 7, 11 and 14 days, and extraction/analysis procedure was applied (Section 2.2, Section 2.3 and Section 2.4). The samples of dry lovage roots (manufacturer Evalar, CJSC; production year 2016; 1 kg) were deposited into the individual polystyrene bags (2 L) and incubated at 10 °C in a ventilated MK 53 thermostat (BINDER GmbH, Tuttlingen, Germany) for 6 years. Two-hundred portions of stored sample were taken out for analysis every year and extraction/analysis procedure was applied (Section 2.2, Section 2.3 and Section 2.4).

### 2.6. Statistical Analysis

Statistical analyses were performed by one-way analysis of variance, and the significance of the mean difference was determined by Duncan’s multiple range test. Differences at *p* < 0.05 were considered statistically significant. The results are presented as the mean ± S.D. The linear regression analysis and generation of calibration graphs were conducted using Advanced Grapher 2.2 (Alentum Software, Inc., Ramat-Gan, Israel).

## 3. Results and Discussion

### 3.1. Phenolic Metabolite Profiling of Fresh Lovage Roots

**Figure 1:**
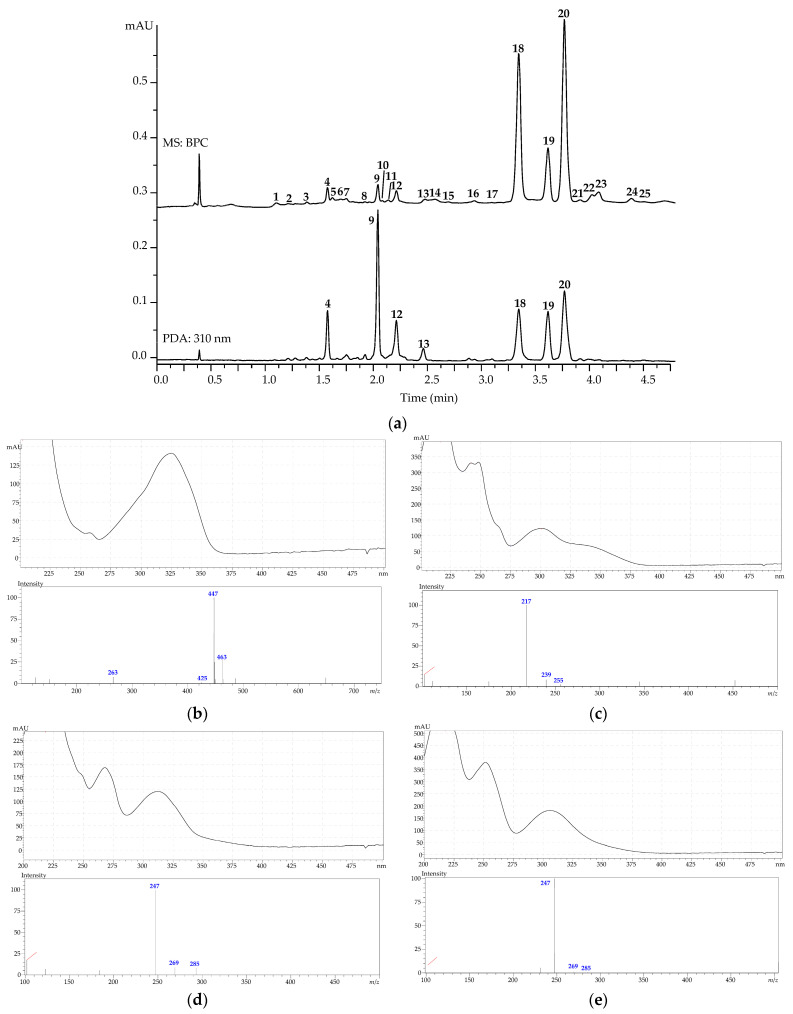
(**a**) High-performance liquid chromatography with electrospray ionization triple quadrupole mass spectrometric detection chromatogram (base peak intensity chromatogram, positive ionization; MS: TIC) and photodiode array detection at 310 nm (PDA: 310 nm) chromatograms of an extract of *L. officinale* fresh roots (cv. Lider). (**b**) UV and mass spectra of principal coumarins: apterin (**b**), xanthotoxin (**c**), isopimpinellin (**d**), and pimpinellin (**e**). Compounds are numbered as listed in Table 2.

Application of HPLC–PDA–ESI–tQ–MS and micro-sized column (1 × 50 mm) successfully separated 25 metabolites in 5 min in fresh lovage root extract. Identification was achieved after comparison of retention times and ultraviolet (UV) and mass spectra with reference standards and the literature data [51,52,53,54,55,56,57,58,59,60,61,62] (Figure 1a and Table 2). The most abundant group of metabolites was coumarins, including nineteen compounds (**1**–**3**, **5**, **6**, **9**, **10**, **14**–**25**), and a lesser group of hydroxycinnamates consisted of six acids (**4**, **7**, **8**, and **11**–**13**). Coumarins have specific absorbance in the UV region [60] and gave a typical triplet in the positive ionization mass spectra, featuring signals of protonated ion [M+H]^+^ and 23 and 39 amu larger adducts with sodium [M+Na]^+^ and potassium [M+K]^+^ [38] (Figure 1b–e).

**Figure 2:**
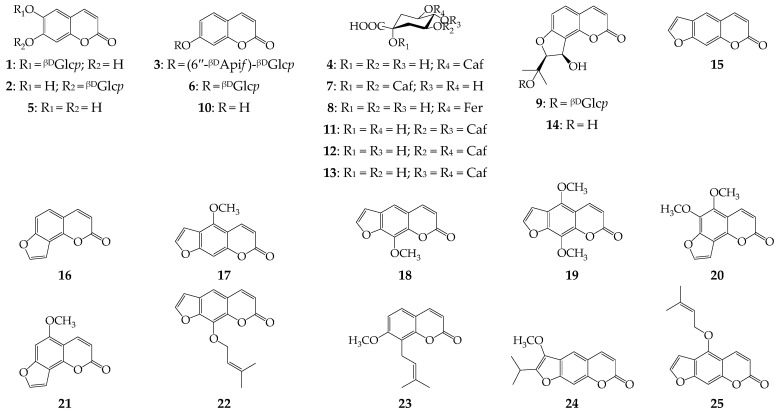
Compounds **1**–**25** found in fresh lovage roots. ^βD^Api*f*–β-D-apiofuranose; Caf–caffeoyl; Fer–feruloyl; ^βD^Glc*p*–β-D-glucopyranose.

Simple coumarins with a bicyclic structure included esculetin (6,7-dihydroxycoumarin, **5**) and two of its glycosides, i.e., of esculin (esculetin 6-*O*-glucoside, **1**) [51] and cichoriin (esculetin 7-*O*-glucoside, **2**) [52]; umbelliferone (7-hydroxycoumarin, **10**) and two of its glycosides, i.e., skimmin (umbelliferone *O*-glucoside, **6**) [55] and apiosylskimmin (**3**) [39]; and osthole (7-methoxy-8-isopentenylcoumarin, **23**) [61] (Figure 2). None of the simple coumarins were previously found in the lovage roots. Eleven furanocoumarins have a non-glycosidic nature with the 2′,3′:7,6-coupled furan ring (e.g., psoralen (**15**) [57], bergapten (**17**) [58], xanthotoxin (**18**) [59], isopimpinellin (**19**) [60], imperatorin (**22**) [60], peucedanin (**24**) [62], and isoimperatorin (**25**) [60]) or the 2′,3′:8,7-coupled furan ring (e.g., vaginol (**14**) [56], angelicin (**16**) [57], pimpinellin (**20**) [60], and isobergapten (**21**) [60]). The only glycosidic furanocoumarin was vaginol 8-*O*-glucoside or apterin (**9**) [56]. Psoralen and imperatorin were previously detected in lovage fruits [28], bergapten was detected in the roots [4], and apterin was detected in the leaves of the plant [14,15]. Compounds **14**, **16**, **18**, **19**, **20**, **21**, **24**, and **25** were found in *L. officinale* for the first time. The known dietary coumarins in rooting apiaceous foods have been found in carrot as 6-methoxymellein [63], bergapten, isopimpinellin, and xanthotoxin [64]; they have been found in fennel as bergapten, isopimpinellin, and xanthotoxin [64]; and they have been found in celery and parsley as umbellifereone, scopoletin and esculetin [34]. Thus, it is clear why lovage roots are able to accumulate coumarins with various structures.

Non-coumarin metabolites of fresh lovage roots are derivatives of caffeic acid as mono- (**4**) and di-*O*-caffeoylquinic acids (**7**, **11**–**13**) as well as 5-*O*-feruloylquinic acid [53]. Acid **4** has been previously found in the herbal part of lovage [12,14,15,31], and the remaining phenolics have been discovered for the first time in *L. officinale*. A previous report [29] showed that some flavonoid compounds can be detected in lovage roots; however, in our case, no member of this group was found. 

### 3.2. Quantification of Four Principal Coumarins in Lovage Roots

Chromatographic conditions applied for metabolite profiling of fresh lovage roots gave appropriate separation of four principal coumarins with more abundant peak areas, such as for apterin (peak **9**), xanthotoxin (peak **18**), isopimpinellin (peak **19**), and pimpinellin (peak **20**), enabling their use for quantification of the mentioned coumarins in plant samples. To simplify and lower the cost of the assay, in this study, PDA detection was used, resulting in a fast and easy method of analysis. The validation procedure demonstrated the good linearity of the calibration equations built for four coumarins with correlation coefficients (r^2^) of 0.9925–0.9981 and standard deviations of (*S*_YX_) 9.76–11.52 × 10^−2^ (Table 3). 

The limits of detection and limits of quantifications were 0.18–0.26 µg/mL and 0.56–0.78 µg/mL, respectively, and the linear range was 0–1000 µg/mL. The intra-day and inter-day precisions were high and showed relative standard deviations (RSDs) of 0.96–1.20% and 1.40–1.93%, respectively, and spiked samples demonstrated high recovery levels from 98.51% to 101.70%. All these results showed the appropriateness of the method for quantification of the principal coumarins in lovage roots.

Approbation of the quantification method was conducted on the fresh samples of seven lovage cultivars and eight commercially available dried lovage roots (Table 4). The contents of apterin, xanthotoxin, isopimpinellin, and pimpinellin varied in fresh roots as 197–357, 152–352, 486–863, and 904–1296 μg/g, respectively, showing that pimpinellin was the most common coumarin in all lovage samples. The total coumarin content in fresh roots ranged from 1739 (cv. Heracles) to 2902 μg/g (cv. Lider). Dried lovage roots demonstrated wide variation of apterin, xanthotoxin, isopimpinellin, and pimpinellin, with values of 1.53–4.11, 1.40–3.75, 4.83–7.80, and 7.36–11.26 mg/g, respectively, and pimpinellin was a common coumarin found in all samples. The range of total coumarin content in dried roots was 15.12–24.46 mg/g. Thus, fresh and dried lovage roots are rich sources of coumarins. 

Regarding the bioactive properties of lovage coumarins, apterin has been previously determined to be a common apiaceous coumarin [65] and showed antioxidant [66], anti-tumor [67], anticholinesterase [68], antidiabetic [69,70], and anti-inflammatory activities [71]. Xanthotoxin has demonstrated various bioactivities, such as anticancer, anti-inflammatory, antioxidative stress and antibacterial activities [72], while pimpinellin was determined to be an effective preventer of platelet-related thromboembolic diseases (such as atherosclerosis [73]), and isopimpinellin is antibacterial agent against methicillin-resistant *Staphylococcus aureus* [74]. Thus, lovage roots are a valuable source of bioactive coumarins.

### 3.3. Post-Harvest Changes in Four Principal Coumarins in Lovage Roots

**Figure 3:**
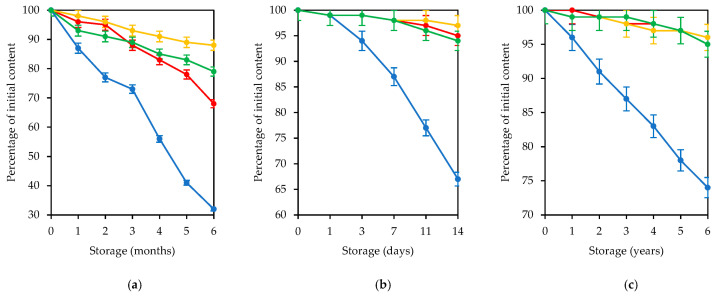
Changes in coumarin content in lovage roots during storage of fresh roots at 1 °C (**a**) and at 20 °C (**b**) and dried roots at 10 °C (**c**). Lines: apterin (blue), xanthotoxin (red), isopimpinellin (yellow), and pimpinellin (green).

Traditionally, the methods of lovage root storage have been similar to those of other apiaceous root (carrot, parsley, celery, and fennel). The best preservation has been observed for chilled storage when the temperature is close to zero; however, room temperature storage is popular for fresh roots. Therefore, postharvest changes in fresh lovage roots were studied under two conditions: one group of samples was stored at 1 °C for 6 months, and second group was conditioned at 20 °C for two weeks. These periods were chosen taking into consideration the satisfactory appearance of vegetables; as a rule, after these dates, roots became flabby (lost firmness) and were no longer stored. Additional study was focused on the changes in dry lovage roots over long-term storage for 6 years at 10 °C (the temperature of a dry plant repository). Studies of the two different types of samples were due to the widespread use of both fresh and dried lovage roots for which it is necessary to determine the composition of coumarins before and after storage (Table 5 and Figure 3).

Chilled storage of fresh lovage roots negatively affected the total coumarin content. Storing roots for 6 months resulted in a loss of 25% of total coumarins, mostly because glycoside apterin losses resulted in 67% damage. Reduction in the content of non-glycosidic xanthotoxin, isopimpinellin, and pimpinellin was no more than 30% of the initial level. Postharvest changes occurred much more rapidly when fresh lovage roots were stored at room temperature. After 2 weeks of storage, a 32% loss of apterin was observed with almost full preservation of other coumarins. Dried lovage roots demonstrated good stability of coumarin content upon long-term storage. Non-glycosidic compounds were resistant and demonstrated approximately a 5% loss after 6 years of storage, and the decrease in glycoside apterin was more than 25%. Despite the loss of compounds during all types of storage, the lovage roots remained a source of coumarins even at the end of the expiration date.

The general trend of postharvest changes in both fresh and dried lovage roots is the significant loss of the glycosidic coumarin apterin. The same changes were observed for other storing plants. The roots of *Hansenia forbesii* (H.Boissieu) Pimenov and Kljuykov (syn. *Notopterygium forbesii* H.Boissieu) can lose up to 60% of the coumarin glycoside nodakenin (nodakenetin *O*-glucoside) during storage while maintaining non-glycosidic compounds [75]. Scopolin (scopoletin *O*-glucoside) and scopoletin reduction was observed in cassava roots (*Manihot esculenta* Crantz) after 7 days of dark storage at 29 °C [76]. Instability of flavonoid glycosides during short- and long-term storage was found for apple [77] and strawberry fruits [78,79]. The possible reasons are increasing cleavage processes that involved the influence of water, acids, and enzymes, resulting in hydrolysis of storage compounds [80]. However, in the case of lovage roots, most coumarins are found in the non-glycosidic form, thus preserving the valuable potential of the plant.

## 4. Conclusions

This study for the first time elucidated the phenolic profile of fresh lovage roots, a traditional food product that is still scarcely investigated. The basic components were simple coumarins and furanocoumarins with various structures with or without glycosidic fragments. Even though some metabolites were in the lovage roots, most identified compounds were new for the *Levisticum officinale* species. Successful chromatographic separation of the principal compounds resulted in creation of a convenient assay for quantification of four coumarins (i.e., apterin, xanthotoxin, isopimpinellin, and pimpinellin), which were found at high levels in both the various cultivars of the fresh lovage roots and in the dry commercial roots. These findings suggest for the first time that lovage roots are a good source of furanocoumarins with proven bioactivity, making lovage a functional food product. The results of the postharvest stability study of lovage coumarins demonstrated a gradual decrease in target compounds, especially the glycoside apterin in fresh and dried roots. However, the final losses accounted for less than one quarter of the total coumarin content, which confirmed satisfactory retention of furanocoumarins in lovage during postharvest storage. Therefore, the known spicy-aromatic vegetable lovage roots accumulate not only phthalides and volatile compounds but also coumarins, making it one of the most valuable apiaceous plants used as food and medicine

## Figures and Tables

**Table 1:** Synopsis of known lovage (*Levisticum officinale*) metabolites.

Compounds	Source [Ref.]
Phtalides	
Butyl phtalide	Roots [4,5], fruit, leaves, stems [5,6], flowers [6]
Butyl dihydrophtalide	Roots [4]
Butyl tetrahydrophtalide	Roots [4]
(*Z*)- and (*E*)-Butylidene phthalide	Roots [4,7,8,9,10], leaves, stems, fruits, flowers [5,6,11,12]
Butylidene 4,5-dihydrophthalide	Roots, fruit, leaves, stems [5]
Propylidene phthalide	Roots [9]
(*Z*)- and (*E*)-Ligustilide	Roots [7,9,10,13], leaves, stems, fruits, flowers [6,11,12,14,15]
7-Methoxy-3-propylidenephthalide	Roots [16]
5-Hydroxybutylidene phthalide	Roots [16]
7-Hydroxybutylidene phthalide	Roots [16]
Senkyunolide	Roots [9]
Isosenkyunolide	Roots [9]
Validene-4,5-dihydrophthalide	Roots [9], leaves, stems, fruits, flowers [6]
Sedanolide	Leaves, stems [6]
Polyynes	
Falcarindiol	Roots [10,13,17]
Falcarinol	Roots [17,18]
Terpenes	
Monoterpenes and sesquiterpenes of essential oils	Roots [5,6,8,9,11,18,19,20,21,22,23,24], fruits [5,6,11,23,25], leaves [5,6,11,12,18,21,26], stems [5,6,11,12,18], flowers [23,24,25,27]
Phenols	
Eugenol	Roots [4]
Carvacrol	Roots [4]
Methyl salycilate	Fruits [6]
Cuminaldehyde	Fruits [25]
Estragole	Roots [11]
Pentyl benzene	Leaves, roots [21]
Coumarins	
Psoralen	Fruits [28]
5-Methoxypsoralen	Fruits [28]
Imperatorin	Fruits [28]
Bergapten	Roots [4]
Apterin	Leaves [14,15]
Phenolic acids	
Gallic acid	Roots [29]
Vanillic acid	Roots [30]
Hydroxycinnamates	
*p*-Coumaric acid	Roots [29]
Caffeic acid	Roots [29], leaves [14,15]
Ferulic acid	Roots [29]
3-*O*-, 4-*O*-, 5-*O*-Caffeoylquinic acids	Leaves, stems [12,14,15,31]
Caffeoylglucaric acid	Roots [30]
Coniferyl ferulate	Roots [10]
Flavonoids	
Kaempferol	Roots [29]
Quercetin	Roots [29]
Myricetin	Roots [29]
Nicotiflorin	Leaves, stems [12]
Isoquercitrin	Roots [29]
Rutin	Roots [29], leaves, stems [12,14,15]
Maclurin-3-*C*-glucoside	Leaves, stems [12]
Other groups	
Catalpol	Roots [30]
Sedanonic anhydride, butyric acid, palmitic acid	Roots [4]
Angeolide	Roots [13]
Nutrients, fatty acids, tocopherols	Leaves, stems [12]

**Table 2:** Chromatographic (*t*), ultraviolet (UV) and mass-spectrometric data (MS) of compounds **1**–**25** found in *L. officinale* fresh roots.

No.	*t*, min	UV, λ_max_, nm	[M+H]^+^, *m*/*z*	[M+Na]^+^, *m*/*z*	[M+K]^+^, *m*/*z*	Other Ions, *m*/*z*	Compound [Ref.]
**1**	1.124	350	341 (5)	363 (100)	379 (32)	179 (9)	Esculin (esculetin 6-*O*-glucoside) [51]
**2**	1.243	348	341 (3)	363 (100)	379 (24)	179 (2)	Cichoriin (esculetin 7-*O*-glucoside) [52]
**3**	1.376	258, 312	457 (7)	479 (100)	495 (38)	163 (14)	Apiosylskimmin [39]
**4**	1.567	240, 327	355 (100)			193 (30)	5-*O*-Caffeoylquinic acid [53]
**5**	1.624	258, 298, 352	179 (100)	201 (7)	217 (4)		Esculetin [54]
**6**	1.687	258, 312	325 (1)	347 (100)	363 (29)	163 (8)	Skimmin (umbelliferone *O*-glucoside) [55]
**7**	1.752	240, 327	517 (100)			355 (42), 193 (2)	1,3-Di-*O*-caffeoylquinic acid [53]
**8**	1.942	238, 321	369 (100)			195 (20), 191 (18)	5-*O*-Feruloylquinic acid [53]
**9**	2.042	258, 324	425 (2)	447 (100)	463 (27)	263 (11)	Apterin (vaginol 8-*O*-glucoside) [56]
**10**	2.084	322	163 (100)	185 (14)	201 (12)		Umbelliferone [55]
**11**	2.127	240, 329	517 (100)			355 (38), 193 (4)	3,4-Di-*O*-caffeoylquinic acid [53]
**12**	2.208	240, 329	517 (100)			355 (37), 193 (1)	3,5-Di-*O*-caffeoylquinic acid [53]
**13**	2.458	240, 329	517 (100)			355 (40), 193 (1)	4,5-Di-*O*-caffeoylquinic acid [53]
**14**	2.567	254, 326	263 (100)	285 (15)	301 (7)		Vaginol [56]
**15**	2.693	244, 298, 334	187 (100)	209 (5)	225 (1)		Psoralen [57]
**16**	2.942	245, 304	187 (100)	209 (10)	225 (3)		Angelicin [57]
**17**	3.083	250, 270, 311	217 (100)	239 (11)	255 (7)		Bergapten [58]
**18**	3.342	242, 251, 301	217 (100)	239 (5)	255 (1)		Xanthotoxin [59]
**19**	3.621	268, 312	247 (100)	269 (21)	285 (18)		Isopimpinellin [60]
**20**	3.759	251, 306	247 (100)	269 (4)	285 (1)		Pimpinellin [60]
**21**	3.882	251, 270	217 (100)	239 (25)	255 (20)		Isobergapten [60]
**22**	4.012	351, 301	271 (100)	293 (18)	309 (10)		Imperatorin [60]
**23**	4.086	256, 324	245 (100)	267 (11)	283 (7)		Osthole [61]
**24**	4.382	255, 294, 341	259 (100)	281 (7)	297 (3)		Peucedanin [62]
**25**	4.501	252, 309	271 (100)	293 (5)	309 (2)		Isoimperatorin [60]

**Table 3:** Regression equations, correlation coefficients (r^2^), standard deviation (*S*_YX_), limits of detection (LOD), limits of quantification (LOQ), and linear ranges for four reference standards.

Compound	a ^a^	b ^a^	Correlation Coefficient (r^2^)	S_YX_	LOD/ LOQ (µg/mL)	Linear Range (µg/mL)	RSD% (Intra-Day)	RSD% (Inter-Day)	Recovery of Spiked Sample REC%
Apterin	1.4400	−0.5261	0.9925	10.14 · 10^−2^	0.23/0.70	0–1000	1.20	1.59	101.70
Xanthotoxin	1.7207	−0.0152	0.9981	9.76 · 10^−2^	0.18/0.56	0–800	0.96	1.40	98.92
Isopimpinellin	1.9320	−0.2419	0.9953	11.52 · 10^−2^	0.20/0.60	0–800	1.07	1.73	99.12
Pimpinellin	1.2844	−0.2915	0.9962	10.02 · 10^−2^	0.26/0.78	0–800	1.14	1.93	98.51

^a^ Calibration equation parameters: y = a · x + b.

**Table 4:** Content of four coumarins in fresh and dried lovage roots.

Plant Source	Apterin	Xanthotoxin	Isopimpinellin	Pimpinellin	Total
Fresh roots, μg/g fresh weight ± S.D.					
cv. Amur	357 ± 7	307 ± 6	683 ± 14	1152 ± 23	2499
cv. Don Juan	289 ± 5	273 ± 5	592 ± 11	1062 ± 21	2216
cv. Heracles	197 ± 4	152 ± 4	486 ± 10	904 ± 18	1739
cv. Lider	391 ± 7	352 ± 7	863 ± 17	1296 ± 25	2902
cv. Magnus	326 ± 6	286 ± 5	794 ± 15	1272 ± 25	2678
cv. Preobrazhenskii	321 ± 6	292 ± 5	837 ± 16	1110 ± 22	2560
cv. Udalets	350 ± 7	311 ± 6	783 ± 15	993 ± 19	2437
Dried roots, mg/g dry weight ± S.D.					
A Gift from Nature Comp. (Orlando, FL, USA)	2.83 ± 0.05	2.96 ± 0.05	7.41 ± 0.14	11.26 ± 0.22	24.46
Alpine Herb Company Inc. (Scarborough, ON, Canada)	1.93 ± 0.03	2.53 ± 0.05	5.72 ± 0.11	8.63 ± 0.17	18.81
Evalar, CJSC (Biysk, Russia)	3.83 ± 0.07	3.25 ± 0.06	6.49 ± 0.12	10.68 ± 0.21	24.25
Khorst, LLC (Barnaul, Russia)	3.26 ± 0.06	2.83 ± 0.05	5.73 ± 0.11	9.14 ± 0.18	20.96
Lekra-Set, LLC (Barnaul, Russia)	4.11 ± 0.08	3.75 ± 0.07	5.37 ± 0.10	10.06 ± 0.20	23.29
Russkie Korni Comp. (Korolyov, Russia)	3.70 ± 0.07	3.14 ± 0.06	5.26 ± 0.10	9.47 ± 0.18	21.57
Starwest Botanicals, Inc. (Sacramento, CA, USA)	4.06 ± 0.08	3.52 ± 0.07	7.80 ± 0.15	8.26 ± 0.16	23.64
TerraVita Comp. (Wilmington, DE, USA)	1.53 ± 0.03	1.40 ± 0.02	4.83 ± 0.09	7.36 ± 0.14	15.12

**Table 5:** Content of four coumarins in fresh and dried lovage roots during post-harvest storage.

Storage Duration	Apterin	Xanthotoxin	Isopimpinellin	Pimpinellin	Total
	Fresh roots (cv. Lider), μg/g fresh weight ± S.D.				
Before storage	391 ± 7	352 ± 7	863 ± 17	1296 ± 25	2902
Months	Chill storage (1 °C)				
1	342 ± 7 *	341 ± 7	854 ± 17	1215 ± 24 *	2752
2	304 ± 6 *	337 ± 7 *	832 ± 16	1183 ± 23 *	2656
3	286 ± 6 *	311 ± 6 *	806 ± 16 *	1157 ± 23 *	2560
4	221 ± 4 *	293 ± 6 *	782 ± 15 *	1102 ± 22 *	2398
5	163 ± 3 *	276 ± 5 *	774 ± 15 *	1083 ± 21 *	2296
6	127 ± 2 *	242 ± 5 *	764 ± 15 *	1026 ± 20 *	2159
Days	Room storage (20 °C)				
1	388 ± 7	350 ± 7	862 ± 17	1290 ± 25	2890
3	370 ± 7 *	348 ± 6	860 ± 17	1283 ± 23	2861
7	342 ± 6 *	345 ± 7	853 ± 18	1272 ± 25	2812
11	304 ± 7 *	340 ± 7	850 ± 16	1254 ± 24	2748
14	265 ± 5 *	332 ± 7 *	842 ± 18	1231 ± 24 *	2670
	Dried roots (Evalar, CJSC), mg/g dry weight ± S.D.				
Before storage	3.83 ± 0.07	3.25 ± 0.06	6.49 ± 0.12	10.68 ± 0.21	24.25
Years	10 °C storage				
1	3.70 ± 0.07	3.25 ± 0.06	6.45 ± 0.12	10.65 ± 0.20	24.05
2	3.52 ± 0.07 *	3.22 ± 0.06	6.42 ± 0.12	10.60 ± 0.21	23.76
3	3.34 ± 0.06 *	3.21 ± 0.06	6.38 ± 0.11	10.58 ± 0.21	23.51
4	3.19 ± 0.06 *	3.19 ± 0.06	6.35 ± 0.12	10.54 ± 0.20	23.27
5	3.02 ± 0.06 *	3.16 ± 0.06	6.30 ± 0.11	10.40 ± 0.20	22.88
6	2.86 ± 0.05 *	3.10 ± 0.06 *	6.25 ± 0.11 *	10.22 ± 0.20 *	22.43

Asterisk indicates significant difference (*p* < 0.05) vs. before storage level.

## Data Availability

The data presented in this study are available in the article.
